# Linezolid-Resistant *Staphylococcus cohnii,* Greece

**DOI:** 10.3201/eid1501.080769

**Published:** 2009-01

**Authors:** Efi Petinaki, Maria Kanellopoulou, Anthi Damani, Antigoni Foka, Iris Spiliopoulou, Nikoletta Skalmoutsou, Bogdan Raitsiou, Konstantinos Valakis, Evangelos Papafragas

**Affiliations:** University of Thessalia, Larissa, Greece (E. Petinaki); Sismanoglion General Hospital of Athens, Athens, Greece (M. Kanellopoulou, N. Skalmoutsou, B. Raitsiou, K.Valakis, E. Papafragas); Institute of BioMedical Research and Technology, Larissa (E. Petinaki, A. Damani); University of Patras, Patras, Greece (A. Foka, I. Spiliopoulou)

**Keywords:** Outbreak, linezolid, Staphylococcus cohnii, Greece, letter

**To the Editor:** Since 2003, linezolid has typically been used to treat infections caused by multidrug-resistant gram-positive cocci such as vancomycin-resistant *Enterococcus faecium* and methicillin-resistant *Staphylococcus aureus* (*1*)*.* In Greece, a major problem is nosocomial dissemination of vancomycin-resistant enterococci. Use of linezolid for the treatment of such infections led to the emergence of linezolid–vancomycin resistant *E. faecium*; however, linezolid resistance of staphylococci is still relatively low in this country (*2*)*.* We describe an outbreak caused by a linezolid-resistant *S*. *cohnii* in an intensive care unit (ICU) in Greece.

From July through October 2007, nonrepetitive coagulase-negative staphylococci that exhibited resistance to linezolid were isolated from blood cultures from 4 separate patients hospitalized in the ICU at Sismanoglion General Hospital of Athens, a 450-bed tertiary care hospital. The ICU is a 10-bed, level II unit, comprising 2 rooms with 1 bed each and 2 rooms with 4 beds each. Each isolate was recovered in 2 of 2 blood culture sets per patient, indicating true bacteremia. The demographic and clinical information for the patients is described in the Table. The mean duration of time preceding linezolid therapy was 22 days.

**Table:** Clinical characteristics of 4 patients from whom linezolid-resistant *Staphylococcus cohnii* was isolated, Greece, 2007*

Patient no.	Gender/ age, y	Medical history	Reason for hospitalization	Previous treatment	Date of hospital admission	Date of sample collection	Outcome	Date of death or discharge
1	F/82	No relevant history	Acute cholecystitis, necrotizing pancreatitis	CIP, CAZ, IMP, TZP, LIN	May 10	Jul 20	Death	Aug 21
2	M/38	Alcoholism	Lung abscess	CIP, CAZ, LIN	Jul 14	Aug 21	Recovery	Oct 20
3	M/52	COPD, melitensus diabetes	Necrotizing pneumonia	TZP, CLI, IMP, LIN	Aug 21	Sep 25	Recovery	Oct 30
4	M/65	No relevant history	Septic shock	IMP, CIP, TZP, LIN	Sep 17	Oct 28	Recovery	Dec 10

*CIP, ciprofloxacin; CAZ, ceftazidime; IMP, imipenem; TZP, piperacillin-tazobactam; LIN, linezolid; CLI, clindamycin; COPD, chronic obstructive pulmonary disease.

Isolates were first identified to the species level by using an API Staph system (bioMérieux, la Balme les Grottes, France) and a molecular method based on the *tuf* gene followed by sequencing analysis (*3*)*.* Susceptibility testing for various antimicrobial agents was performed by disk diffusion and using Clinical Laboratory Standards Institute criteria; susceptibilities were interpreted according to Institute guidelines (*4*)*.* In addition, MICs to oxacillin, vancomycin, teicoplanin, quinupristin-dalfopristin, linezolid, daptomycin, and tigecycline were determined by Etest (AB Biodisk, Solna, Sweden) according to manufacturer’s instructions. Resistance genes *mecA****,*** *vat****,*** *vga, erm, aac(6´)-Ie+aph(2″)*, *ant(4′)-Ia*, and *aph(3′)-IIIa*, as markers for resistance to β-lactams, dalfopristin, macrolides, and aminoglycosides, were identified by PCR as previously reported (*5*,*6*). The presence of G2576T in domain V of the 23S rRNA, which is mainly associated with linezolid resistance in clinical isolates, was detected by using PCR and digestion of the product with *NheI* (*2*)*.* The number of mutated versus nonmutated alleles was determined as described by Pillai et al. (*7*)*.* In addition, isolates were examined for the presence of the *cfr* gene, which was found to be correlated with linezolid resistance in some coagulase-negative staphylococci and for mutations of ribosomal protein L4, L22 genes (*8**,**9*)*.* Clonality of isolates was assessed by pulsed-field gel electrophoresis (PFGE) after digestion of chromosomal DNA with *SmaI* (*2*)*.*

The molecular method identified the isolates as *S. cohnii* subsp. *ureolyticus*. The API Staph system has correctly identified 2 of them; the remaining 2 isolates were falsely characterized as *S. xylosus*. According to disc diffusion test results, the isolates were resistant to cefoxitin, oxacillin, penicillin, rifampin, quinupristin-dalfopristin, erythromycin, clindamycin, fusidic acid, tobramycin, gentamicin, and linezolid. MICs were linezolid 32, oxacillin 256, quinupristin-dalfopristin 8, vancomycin 2, teicoplanin 2, tigecycline 0.125, and daptomycin 0.5 mg/L. Molecular methods detected the following resistance genes: *mecA, ermA,* *aac(6´)-Ie+aph(2″),* and *aph(3´)-IIIa.* The isolates, despite their resistance to streptogramins, were negative for *vat* and *vgaA* genes. In addition, all isolates carried the G2576T mutation and had 4 of 5 mutated alleles. No isolate carried the *cfr* gene or any mutation on ribosomal protein L4 and L22 genes. PFGE results indicated that all isolates were clonally related, belonging to the same clone.

Outbreaks caused by linezolid-resistant staphylococci are rare worldwide (*10*); in Sismanoglion Hospital, before the outbreak period, no linezolid-resistant staphylococci and enterococci had been isolated. However, in the ICU, a statistically significant increase in the use of linezolid was observed in 2004 and in 2007 (0.58 vs. 1.34 defined daily doses/100 patient-days, respectively); heavy use of linezolid may have created substantial selection pressure in favor of the linezolid-resistant isolates.

The 4 patients were treated in the same room by the same personnel; thus, a potential explanation for this outbreak is patient-to-patient transmission of linezolid-resistant strains on the hands of healthcare personnel. However, cultures of ICU personnel (nasal cavity and hands) grew only methicillin-resistant *S. aureus* and methicilllin-resistant *S. epidermidis*. In addition, environmental samples taken from the beds and the equipment of these patients were negative for *S. cohnii*. Strict control measures were taken (e.g., isolation of infected patients, increased environmental cleaning, and reinforcement of proper glove and gown use and hand disinfection with alcohol gel), and the outbreak strain was not recovered from other patients in the ICU or in other departments of the hospital after the initial outbreak. In conclusion, to avoid spread of staphylococcal resistance in ICUs, measures such as hand hygiene and adequate central venous catheter handling should be taken, and policies regarding antimicrobial drug use must be applied.
